# Draft Genome Sequence of Pseudomonas sp. Strains MWU12-2319 and MWU12-2311, Isolated from a Wild Cranberry Bog in the Cape Cod National Seashore

**DOI:** 10.1128/mra.00869-22

**Published:** 2022-10-12

**Authors:** Arin Pittman, Scott Soby

**Affiliations:** a Arizona College of Osteopathic Medicine, Midwestern University, Glendale, Arizona, USA; b Biomedical Sciences, College of Graduate Studies, Midwestern University, Glendale, Arizona, USA; c College of Veterinary Medicine, Midwestern University, Glendale, Arizona, USA; University of Arizona

## Abstract

Pseudomonas sp. strains MWU12-2319 and MWU12-2311 were isolated from the soil of a wild cranberry bog in the Cape Cod National Seashore as part of a culture-dependent bacterial population survey. The genomes exceed 7 Mbp and contain putative gene clusters for the biosurfactant orfamides A and C.

## ANNOUNCEMENT

The genus Pseudomonas contains highly diverse species that are adapted to many different environments, but little is known about their evolution, rate of speciation, or how these bacteria influence the microbiomes with which they interact (1–3). As part of a larger microbiome analysis project, we sampled bacteria from wild cranberry bogs to analyze their genomes for taxonomic placement and to provide further insights into their potential ecological functions. Pseudomonas sp. strains MWU12-2319 and MWU12-2311 were isolated from sandy bog soil in the Cape Cod National Seashore (42.064742N, 70.117562W). Bacteria were isolated on King’s medium B (KMB) agar containing 50 μg mL^−1^ each of cycloheximide and ampicillin, incubated at 26°C for 24 to 48 h, colony purified three times on KMB agar, and stored at −80°C in 34% glycerol. All kits described below were used according to the manufacturer’s instructions. Isolates from frozen storage were recovered on KMB agar, and populations were inoculated into overnight KMB broth cultures for genomic DNA isolation with a DNeasy blood and tissue kit (Qiagen, USA). Illumina-compatible genomic DNA libraries were generated using a HyperPlus library preparation kit (Kapa Biosystems product number KK8514; Roche, USA). DNA was enzymatically sheared to ~500 bp, end repaired, and A-tailed. Illumina-compatible adapters with unique indexes (product number 00989130v2; Integrated DNA Technologies, Coralville, IA) were ligated to each sample. Adapter-ligated molecules were cleaned using KAPA pure beads (Kapa Biosystems product number KK8002) and amplified with KAPA HiFi enzyme (Kapa Biosystems product number KK2502). Library fragment sizes were determined on an Agilent TapeStation system, and fragments were quantified by quantitative PCR (KAPA library quantification kit [Kapa Biosystems product number KK4835]) on a QuantStudio 5 system (Thermo Fisher Scientific, USA) before multiplex pooling and sequencing in a 2 × 250-bp flow cell on the Illumina MiSeq platform. All software was used with default settings except as indicated. Raw reads were assembled with Unicycler v0.4.8 (4) and polished with Pilon v1.23 (5) within the PATRIC Comprehensive Genome Analysis pipeline v3.6.12 using default settings except for the trim setting, which was set to true (6). The Comprehensive Genome Analysis pipeline includes quality control and trimming by QUAST v5.0.2 (7) and Trim Galore v0.4.0 (8) and annotation by RASTtk v1.073 (9), supplemented with antiSMASH (https://antismash.secondarymetabolites.org/#!/start) for recognition of secondary metabolite gene clusters (10). Using the Type (Strain) Genome Server (TYGS) (11), isolates were placed with high confidence within the genus Pseudomonas. Digital DNA-DNA hybridization (dDDH) (formula d4) with Pseudomonas batumici UCM B-321^T^ (GenBank accession number JXDG00000000), the closest relative within the genus, yielded values of 42.1% for both isolates, indicating that MWU12-2319 and MWU12-2311 are not closely related to any named Pseudomonas species. The isolates contain putative gene clusters for synthesis of orfamide A and C, biosurfactant cyclic lipopeptides (CLPs) that have activity against oomycete zoospores and filamentous fungi and insecticidal activity against aphids, as found previously in the Pseudomonas protegens group (12, 13).

### Data availability.

The whole-genome shotgun project has been deposited in DDBJ/EMBL/GenBank under BioProject accession number PRJNA691338, with the genome and SRA accession numbers presented in Table 1. The versions described in this paper are JALJES000000000.1 (MWU12-2319) and JAMQRU000000000.1 (MWU12-2311). RASTtk annotations are available under open license at Zenodo (https://doi.org/10.5281/zenodo.6413223 [MWU12-2319] and https://doi.org/10.5281/zenodo.6629972 [MWU12-2311]).

**TABLE 1:** Data summary

Isolate	BioSample accession no.	GenBank accession no.	SRA accession no.	Genome size (bp)	No. of contigs	*N*_50_ (bp)	G+C content (%)	Total read length (bp)	No. of reads	Coverage (×)
MWU12-2319	SAMN26896722	JALJES000000000	SRR18508975	7,276,095	83	299,807	61.24	716,456,692	3,085,670	98
MWU12-2311	SAMN27783457	JAMQRU000000000	SRR19044811	7,276,098	85	284,877	61.24	975,020,131	4,169,776	134
